# Characterization of Sphenoid Sinuses for Sudanese Population Using Computed Tomography

**DOI:** 10.5539/gjhs.v6n1p135

**Published:** 2013-10-28

**Authors:** Samih A. Kajoak, Caroline Edward Ayad, Elsafi Ahmed Abdalla Balla, Mohammed Najmeldeen, Mohammed Yousif, Alamin Musa

**Affiliations:** 1 College of Medical Radiological Science, Sudan University of Science and Technology, Khartoum, Sudan; 2 Radiology Department, Fedail Hospital, Khartoum, Sudan

**Keywords:** sphenoid sinuses, CT measurement, anatomical variation

## Abstract

The purpose of this study is to determine the anatomical features of the sphenoid sinus using computerized tomography (CT).

100 Sudanese subjects were investigated for CT sinuses; Characterization of the sphenoid sinus and seven horizontal and vertical measurements were evaluated.

Onodi cell was found in 13 subjects, 10 of them were sellar and 3 were pre-sellar. Pneumatization was of the sellar type in 85%, presellar was 15%, and no subject was chonchal.

The mean length of vertical lines from the center of sphenoid ostium to the roof and bottom were 10.6 ± 3.1 mm, 11.1 ± 3.7 mm respectively. When the sphenoid ostium was located superior to the lowest point of the sella, the line from the center of the sphenoid sinus ostium to the posterior wall of the sinus was 15.2 ± 4.2 mm and when was located inferior, the line was 26.3 ± 5.2 mm on average. The mean length from the lowest point of the sella to the anterior wall of sphenoid sinus was 16.8 ± 3.6 mm. The line from anterior wall to posterior wall of sphenoid sinus lining skull base was 10.9 ± 3.2mm mm. The maximum depth was 25.2 ± 6.9 mm and the maximum width was 18.4 ± 5.9mm. The differences in the sphenoid sinus character take place between males and females.

The study provides essential anatomical information for Sudanese subjects and its impact in the clinical surgical practice.

## 1. Introduction

Sphenoid sinuses are the most inaccessible paranasal sinuses and are surrounded by significant anatomical structures; only thin plates of bones separate these structures from the sphenoid sinus (Bademci & Unal, 2005) which can make sphenoid sinus surgeries critical.

The anatomical variation of the sphenoid sinus had been studied (Cheung et al., 1993; Mafee, Chow, & Meyers, 1993; Delano, Fun, & Zinrich, 1996). The nasal anatomy shows different variations including optic and vidian canals that are related to sphenoid sinuses and may predispose the patients to different disease as air flow obstruction, recurrent or chronic sinusitis (Dwivedi & Singha, 2010; Kanlikama, 2000; Bolger, Butzin, & Parsons, 1991).

Computerized tomography is the best imaging technique to demonstrate paranasal sinuses (Kainz & Stammberger, 1992; Arsalan et al., 1997; Zinreich, 1998). Computed tomography (CT) has been routinely used for patients with sinusitis, as well as analysis of paranasal sinus anatomy through scanning.

In order to minimize risks during surgery; a careful analysis should be coupled with knowledge about the anatomical variations on CT scans before surgical intervention. CT scan can help the surgeon to enhance identification of the limits of dissection during surgery. Besides, being aware of the exact knowledge of anatomic morphology and probable anomalies are of particular importance to the surgeon (Bolger et al., 1991).

Anatomical bony and mucosal structures of this region can be demarcated at CT by maximum accuracy (Delano et al., 1996). The axial and coronal planes are very useful in identifying the ostium of sphenoid sinus and Onodi cell. In order to obtain more surgical anatomical information, we must measure the line in sagittal plane through the operation path (Wu et al., 2011).

The anatomy of the sphenoid sinus is complex and its surgery is difficult, to our knowledge no identical study was done for Sudanese in the open literature.

The aims of the study were to show the anatomic variations of sphenoid sinus and related structures for Sudanese, as well as to study the role and impact of sagittal and coronal computerized tomography cuts in identification of the anatomical variations.

## 2. Method

The study was carried out during the period from June2012 up to May 2013 at the Radiology Department of Royal Care Hospital in Sudan. The sample comprised of 100 Sudanese subjects attended for CT scanning for sinuses 56(56%) were males and 44(44%) were females and with ages ranging between (18-90) years. Subjects were diagnosed as normal sinus. Patients having pathological changes as neurological deficit, stroke, epilepsy, vertigo, sinusitis, any congenital abnormalities in sphenoid sinuses and subjects younger than 16 years were excluded.

Multi slice CT scanner (Toshiba Aquilion 64 CT scanner) was used. Axial Paranasal sinus CT, were obtained in parallel projection to the orbitomeatal line, with a (1 mm) slice thickness and a (0.6 mm) reconstruction interval. Three-dimensional images were obtained by reconstruction of the axial images using slice thickness equal to (0.4mm). The measurements were performed on sagittal and coronal images using (4000 HU window width, and 400HU window level); Data were obtained using technical properties of 120 kVp, 150 mAs. The subjects were prepared by giving full Information about the procedure.

The following variants were studied; as well as the type of sphenoid sinus (Sellar – pre-sellar – Conchal) and this was according to the classification of (Hammer & Radberg, 1961). The existence of an Onodi cell in the sphenoid sinus, age, gender had been evaluated. The patients included in the study were evaluated by the same Radiologist. The measurements were obtained with both left and right sides for comparisons.

Measurements relating to sphenoid sinus with its surrounding structures are as follows: all measurements from the sphenoid ostium are from the mid point of sphenoid ostium and are taken in (mm):

- *Line 1*: The distance to the roof of sphenoid sinus.
- *Line 2*: The distance to the bottom of sphenoid sinus.
- *Line 3*: The distance to the posterior wall of (SS).
- *Line 4*: The distance from the bottom of sella to the anterior wall of sphenoid sinus.
- *Line 5*: The skull base length from the anterior wall of sphenoid sinus to the sella.
- *Line 6*: maximum depth.
- *Line 7*: maximum width

All Measurements are performed on sagittal images except in Line 7; the measurements are performed on coronal images. SPSS software version 16.0 was used and for the statistical analysis, ANOVA test, Independent samples T-test, mean and Standard deviation were used.

## 3. Results

The 100 Subjects studied consist of 44(44%) females and 56(56%) males. The mean age of the subjects was 42.8years old ranging between 19 and80 years. The overall mean measurements of the variables were classified according to sphenoid sinuses type as onodi and non- onodi; the values were presented in (Table 1). The results showed a significant difference between non- onodi cell and Onodi cell in lines (1, 4, 5, 6) (P-value < 0.05), but in line (2, 3, 7) there were no significant differences between non -Onodi cell and Onodi cell (P-value > 0.05) (Table 1).

**Table 1:** The measurements of sphenoid sinuses in both Onodi and Non -Onodi type

Lines	Non Onodi Cell	Onodi Cell	Total	P-value
**Line 1**	10.8 ± 3.0	8.9 ± 3.2	10.6 ± 3.1	0.003*
**Line 2**	11.2 ± 3.8	10.1 ± 2.2	11.1 ± 3.7	0.152
**Line 3**	22.9 ± 7.3	20.8 ± 6.1	22.6 ± 7.1	0.162
**Line 4**	17.1 ± 3.5	15.1 ± 4.0	16.8 ± 3.6	0.007*
**Line 5**	11.2 ± 3.2	9.2 ± 2.8	10.9 ± 3.2	0.004*
**Line 6**	25.7 ± 6.9	22.0 ± 5.8	25.2 ± 6.9	0.010*
**Line 7**	18.4 ± 5.8	18.4 ± 7.1	18.4 ± 5.9	0.997

Values are expressed as Mean ± SD

* Significant at P<0.05

The mean length of (Line 1) was 10.6 ± 3.1 mm. There was a significant difference between non -onodi cell and onodi cell in (Line 1), 10.8 ± 3.0 mm in non -onodi cell type and 8.9 ± 3.2 mm in onodi cell type (Table 1). Moreover there was no statistically significant difference in the length of (Line 1) between males and females in both sides (p>0.05) (Table 2). The mean length of Line 2 was 11.1 ± 3.7 mm. There was no significant difference between onodi cell type and non- onodi cell types in (line 2) (Table 1), but there is a significant difference between males and females in left side in (line 2) (p= 0.013) (Table 2).

**Table 2:** The mean and standard deviation and P- value of the seven lines in both gender for both the right and left sides

		Gender	Right side	Left side
***Line 1***		**Female**	10.1 ± 2.8	10.2 ± 3.0
		**Male**	11.1 ± 3.3	10.7 ± 3.2
		**Total**	10.7 ± 3.1	10.5 ± 3.1
		**P-value**	0.134	0.422
***Line 2***		**Female**	10.6 ± 3.5	10.0 ± 3.6
		**Male**	11.5 ± 3.5	11.9 ± 3.9
		**Total**	11.1 ± 3.5	11.1 ± 3.9
		**P-value**	0.201	0.013*
***Line 3***	**Inferior**	**Female**	24.7 ± 5.0	23.9 ± 4.4
		**Male**	28.2 ± 4.9	27.7 ± 5.2
		**Total**	26.6 ± 5.2	26.0 ± 5.2
		**P-value**	0.006*	0.003*
	**Superior**	**Female**	13.9 ± 3.1	14.8 ± 3.4
		**Male**	15.9 ± 3.9	15.8 ± 5.4
		**Total**	15.0 ± 3.7	15.4 ± 4.6
		**P-value**	0.121	0.533
***Line 4***		**Gender**	15.9 ± 3.6	15.6 ± 3.7
		**Female**	17.6 ± 2.9	17.6 ± 3.7
		**Male**	16.8 ± 3.3	16.7 ± 3.9
		**Total**	0.007*	0.018*
***Line 5***		**Female**	10.6 ± 3.3	10.3 ± 3.8
		**Male**	11.4 ± 2.7	11.1 ± 3.2
		**Total**	11.1 ± 3.0	10.8 ± 3.5
		**P-value**	0.151	0.253
***Line 6***		**Female**	23.2 ± 7.1	23.6 ± 6.4
		**Male**	26.4 ± 7.0	27.0 ± 6.4
		**Total**	25.0 ± 7.2	25.5 ± 6.6
		**P-value**	0.028*	0.009*
***Line 7***		**Female**	17.1 ± 5.6	17.2 ± 6.5
		**Male**	18.1 ± 5.1	20.7 ± 6.0
		**Total**	17.7 ± 5.3	19.1 ± 6.5
		**P-value**	0.369	0.007*

Values are expressed as Mean ± SD Standard Deviation:

* Significant at P<0.0

When the sphenoid ostium was located superior to the lowest point of the sella, Line 3 was 15.2 ± 4.2 mm on average. When the sphenoid ostium was located inferior to the lowest point of the sella, the horizontal line described was 26.3 ± 5.2 mm on average (Table 2). There is significant difference between males and females in both right and left sides in (Line 3), when it is located inferior to sella (p= 0.006) and (p= 0.003) respectively, and there is no significant difference between males and females in the right and left sides in (Line 3) when it is located superior to the sella (Table 2) and there was no significant difference between onodi cell type and non -onodi cell types in (Line 3) (Table 1).

Line 4 mean value was 16.8 ± 3.6 mm in length. There was a significant difference between non -onodi cell and onodi cell in (Line 4), 17.1 ± 3.5 mm in non- onodi cell type and 15.1 ± 4.0 mm in onodi cell type (Table 1). Also there was a statistically significant difference in the length of (Line 4) between males and females in both right and left sides, *P=0.007* and *P=*0.018 (table 2). The mean distance of Line 5 was 10.9±3.2mm. There was a significant difference between non- onodi cell and onodi cell in (Line 5), 11.2 ± 3.2 mm in non- onodi cell type and 9.2 ± 2.8 mm in onodi cell type (table 1). In addition, there was no statistically a significant difference in the length of (Line 5) between males and females in both sides (p>0.05). The maximum depth (Line 6) was 25.2 ± 6.9 mm in length. There was a significant difference between non- onodi cell and onodi cell in (Line 6), 25.7 ± 6.9 mm in non- onodi cell type and 22.0 ± 5.8 mm in onodi cell type. Furthermore there was a significant difference between males and females in right and left sides in (Line 6), (p = 0.028) (p = 0.009). The maximum width (Line 7) was 18.4 ± 5.9 mm. There was no significant difference between onodi cell type and non- onodi cell types in (line 7) (Table 1), but there is a significant difference between males and females in the left side in (Line 7) (p =0.007) (Table 2).

## 4. Discussion

During fetal development, the paranasal sinuses originate as invagination of the nasal mucosa into facial and cranial bones. This development explains the anatomical variation. Computed tomography (CT) is an excellent means of providing anatomical information of this region (Amit N D Dwivedi & Kapil Kumar Singha, 2010).

In the past decade, and due to the development of diagnostic techniques, the endonasal endoscopic approaches to the sinus and to the latest advancement in surgery, allow using the sphenoid sinus as a tract to the sellar area (Van Alyea OE 1941, Paolo Castelnuovo et al., 2009)

The distance from the sphenoid ostia to the anatomical structures surrounding the sphenoid sinus is important (Alvaro Campero et al., 2010). Therefore, we designed 7 lines to measure the distance of sphenoid sinus, 6 lines in sagittal plane and one line in coronal plane. The transverse and coronal planes are important in the visualization of the sphenoid ostium and onodi cells. To obtain more surgical anatomic information, sagittal plane measurements of the surgical path should be performed.

In our study, the mean length of Line 1 was 10.6 ± 3.1 mm so if the surgeon proceeds to more than the defined height, the skull base might be perforated. There are a similar studies performed by Wu et al. (2011) on a Chinese population, Hatice et al. (2013) on Turkish population and Jae et al. (2012) on Korean population found that the average length of Line 1 was found to be 10.6± 1.5 mm, 7.3±2.4 mm and 9, 97± 3.06 mm consequently, demonstrating that line 1 in Sudanese population has the similar value in Chinese and Turkish population and significant difference with Korean population values. The mean length of (Line 2) was 11.1 ± 3.7 mm. In the study by Wu et al. (2011), the average length of Line 2 was 12 ± 3.7 mm. Hatice et al. (2013) on Turkish population found that line 2 was 12.9 ± 3.8 mm and Jae et al. (2012) on Korean population found that the value of line 2 was 10.44 ± 2.6 mm. All these values were not similar to that obtained in our study, demonstrating that the surgeon should be very aware when he wants to dilate the sphenoid ostium downward, and these differences may be due to Sudanese subjects vary from other populations.

Line 3 according to the localization of the ostium, was 15.2 ± 4.2 mm or 26.3 ± 5.2 mm. According to the distances defined, to avoid the injuries to the sella, sphenoid sinus procedures should not exceed a depth of 15 mm. In the study by Wu et al. (2011), the average length of Line 3, according to the localization of the ostium, was 18 ± 1.5 mm or 28± 2.5 mm. Hatice et al. (2013) on Turkish population found that line 3 was 14.4±3.8 mm or 24.2±5.4 mm and Jae et al (2012) on Korean population found that the value of line 3 was 13.44 ± 3.27 mm or 20.30± 7.63. The risk of injury to the sella was found to be greater in the Sudanese, Korean and Turkish populations than in the Chinese populations. If the sphenoid ostium is localized superior to the lowest point of the sella, the surgeon should be very careful during the procedure (Kim et al., 2001; Kieff & Busaba, 2002). When entering the sphenoid sinus, the unique borderline for guiding the surgeon into the depths of the sinus and finding the targeted point is the posterior wall of the sphenoid sinus, although the risk of injury to the pituitary gland is quite high (Li et al., 2008). If the sinus ostium is localized inferior to the lowest point of the sella, then when entering the sphenoid sinus, the surgeon will immediately come across the posterior wall of the sinus.

Line 4 was 16.8 ± 3.6 mm. In the study by Wu et al. (2011), the average length of Line 4 was found to be 17.5±1.3 mm. Hatice et al. (2013) on Turkish population found that line 4 was 19.09± 3.5 mm and Jae et al. (2012) on Korean population found that the value of line 4 was 10.44 ± 2.6 mm, which was the shortest value comparing with other population. Line 5 was 10.9±3.2mm, Wu et al. (2011) in their study found that the average length of Line 5 was 10.1 ±1.0 mm. Hatice et al. (2013) on Turkish population found that line 5 was 10.8 ± 5.03 mm and Jae et al. (2012) on Korean population found that the value of line 5 was 9.46 ± 4.28 mm. All these values of line 5 is not much different than the length obtained in the present study.

Due to the obliquity of Lines 4 and 5 related to the pituitary fossa, we should be attentive not to wrongly estimate the distance, in order to avoid complications of sella while performing the surgery.

The maximum depth (Line 6) was 25.2 ± 6.9 mm in length. Our results showed that this depth is 2.5 times longer than line 5 and that means the entrance of sphenoid sinus in Sudanese population to drainage of inflammatory processes involving the sphenoid sinus (mucoceles, mucopyoceles, fungal sinusitis, etc.) as well as surgery is safer from below. In the study by Wu et al. (2011), the average length of Line 6 was 22.0 ±7.7 mm. Hatice et al. (2013) on Turkish population found that line 6 was 24.2 ± 6.8 mm and (Jae et al,2012) on Korean population found that the value of line 6 was 25.5 ± 7.55 mm. The maximum width (Line 7) was 18.4 ± 5.9 mm. To our knowledge there was no research done for measuring the width of sphenoid sinus using CT in coronal plane in adult patients. In the study by Barghouth et al. (2002), the average width of sphenoid sinus in children under 17 years old was 12.8± 3.1, using MRI machine.

Studies by Wu et al. (2011), and Jae et al. (2012), revealed that no differences between the left and the right sides and males and females were found concerning the defined distances. On the other hand, in the study by Hatice et al. (2013) found that lines 4 and 6 were longer on the left side than on the right side. Additionally, Lines 2, 3, 4 and 6 were found to be longer in males than in females. However, in the present study, no differences between the left and the right sides were noted but the Lines 4 and 6 found to be longer in males than in females in both sides and the lines 2 and 7 were longer in males than in females only on the left side than on the right side, signifying that gender differences should be considered in sphenoid sinus procedures.

Kainz and Stammberger defined an onodi cell and its relation to the optic nerve and optic canal (Kainz & Stammbergcr, 1991). Its prevalence in Sudanese population is unknown before our study, which showed that there is a significant presence of posterior ethmoid cells in Sudanese population and the surgeons should be aware during surgery because the cerebrospinal fluid leakage is the most common complication (Von Aken et al., 2004; Marc et al., 2006) during transsphenoidal surgery, and the sagittal CT image is necessary before dilatation the sphenoid ostium. In all 100 patients in our study, onodi cells were present in 13 % patients (7 males, 6 females), 10 of them were sellar and 3 were pre-sellar. Onodi cells were absent in (87%) of the patients (Table 3). The presence of onodi cells should be routinely looked for by Functional endoscopic sinus surgery surgeons to prevent damaging the optic nerve. There was a significant difference between non- onodi cell and onodi cell in line (1, 4, 5, 6) (P-value < 0.05), but in line 2, 3, 7 there was no significant difference between non- onodi cell and onodi cell (P-value > 0.05) (Table 1). In onodi cell-positive subjects, the superior wall of the sphenoid sinus did not form the skull bottom; rather, it formed the bottom of the Onodi cells. Line 1 was found to be shorter in Onodi cell-positive patients compared to the Onodi cell-negative patients. In spite of being shorter, the distance in Onodi cell-positive patients is much safer, because during sphenoid surgery, the skull base is not directly bordering to the sphenoid sinus (Wu et al., 2011). In Onodi cell-positive patients, the anterior wall of the sphenoid sinus (Line 5) was located more posteriorly. In such cases, the danger of injury to the sella is not greater compared to the other groups because the sphenoid ostium is located more superior to this level. These results were similar results obtained by Wu et al. (2011), Hatice et al. (2013) and Shin et al. (2012).

**Table 3:** Cross tabulation between the Onodi, Non -Onodi type with Sellar, presellar and conchal with gender

			Gender		Total
			Female	Male
sphenoid sinuses Classification	Sellar	Count	34	51	85
		% within Gender	77.3%	91.1%	85.0%
	Pre-sellar	Count	10	5	15
		% within Gender	22.7%	8.9%	15.0%
	Conchal	Count	0	0	0
		% within Gender	0.0%	0.0%	0.0%
sphenoid sinuses Classification	Onodi cell	Count	6	7	13
		% within Gender	13.6%	12.5%	13.0%
	Non Onodi cell	Count	38	49	87
		% within Gender	86.4%	87.5%	87.0%

Sphenoid sinus has been classified into three types: sellar, presellar, and conchal (Hammer & Radberg, 1961).

In a series of 100 subjects; the research found out that the sphenoid sinuses was of the sellar pneumatization type in 85(85%), males were 51(91.1%) and females were 34(77.3%). Presellar pneumatization was seen in the remaining 15(15%), males were 5(8.9%) and females were 10(22.7%) and no subject was choncal. The onodi type was found in 13(13%) of the subjects, males were 6(13.6%), females were 7(12.5%) where the non- onodi type was found in 87(87%) of the sample 38(86.4%), 49(87.5%) for females and males respectively (Table 3).

## 5. Conclusion

The present data regarding the vertical and horizontal dimensions of sphenoid sinus indicate that the dimensions of sphenoid sinus may anatomically vary. Sudanese population sphenoid sinuses character differs from other studied populations and also the differences in the size of sphenoid sinus take place between males and females. These Anatomic differences of the sphenoid sinus between genders should be taken into consideration during surgery. The present study results show that the pneumatization of the sphenoid sinuses in Sudanese population was sellar and presellar types and no presence of choncal type was found. Using sagittal and coronal CT images is of significant value in characterization of sphenoid sinuses.
